# Cognitive Interventions for Cancer-Related Cognitive Impairment: A Meta-Analysis of RCTs

**DOI:** 10.1093/geroni/igaf122.2901

**Published:** 2025-12-31

**Authors:** Ah Rim Lee, Moonkyoung Park, Moira Visovatti, Kyeongin Cha, Misook Jung

**Affiliations:** Chungnam National University, Daejeon, Republic of Korea; Chungnam National University, Daejeon, Republic of Korea; Eastern Michigan University School of Nursing, Ypsilanti, Michigan, United States; Chungnam National University, Daejeon, Republic of Korea; Chungnam National University, Daejeon, Republic of Korea

## Abstract

Cancer-related cognitive impairment (CRCI) is a common challenge among cancer survivors of both older and younger ages, adversely affecting attention, processing speed, memory, and executive function. These deficits often compromise daily activities and occupational performance. Given the limited efficacy of pharmacological treatments, non-pharmacological cognitive interventions have emerged as promising alternatives, although their effectiveness remains variable. This meta-analysis consolidates current evidence on the efficacy of cognitive interventions for enhancing cognitive function in individuals with CRCI. A systematic search of six databases was conducted to identify randomized controlled trials that evaluated cognitive training interventions for CRCI. Studies were included if they assessed cognitive outcomes using standardized neuropsychological tests and/or self-reported measures. The risk of bias was assessed using Cochrane’s RoB 2.0, and effect sizes were calculated using Hedges’ g with a random-effects model in CMA 4.0. A total of 28 RCTs (n = 1,998) were included, with outcomes from both neuropsychological tests and self-reported measures. The interventions yielded significant improvements in several cognitive domains, including attention (k = 24, g = 0.24), processing speed (k = 19, g = 0.26), verbal memory (k = 22, g = 0.31), visuospatial memory (k = 9, g = 0.33), and executive function (k = 23, g = 0.20). Additionally, subjective cognitive function showed notable improvement (k = 25, g = 0.40). Subgroup analyses revealed that cognitive training (k = 19, g = 0.31) and meditation (k = 7, g = 0.32) produced significant effects, while exercise-based intervention did not yield significant benefits. This meta-analysis provides robust evidence supporting beneficial effects of cognitive interventions for managing CRCI. Further research is needed to identify the optimal training modalities and to assess long-term benefits, which guide clinical practices.

